# Autonomous Oscillatory Mitochondrial Respiratory Activity: Results of a Systematic Analysis Show Heterogeneity in Different In Vitro-Synchronized Cancer Cells

**DOI:** 10.3390/ijms25147797

**Published:** 2024-07-16

**Authors:** Olga Cela, Rosella Scrima, Consiglia Pacelli, Michela Rosiello, Claudia Piccoli, Nazzareno Capitanio

**Affiliations:** Department of Clinical and Experimental Medicine, University of Foggia, 71122 Foggia, Italy; consiglia.pacelli@unifg.it (C.P.); michela.rosiello@unifg.it (M.R.); claudia.piccoli@unifg.it (C.P.); nazzareno.capitanio@unifg.it (N.C.)

**Keywords:** circadian rhythms, mitochondrial respiration, in vitro synchronization, oxygen consumption measurement, Cosinor analysis

## Abstract

Circadian oscillations of several physiological and behavioral processes are an established process in all the organisms anticipating the geophysical changes recurring during the day. The time-keeping mechanism is controlled by a transcription translation feedback loop involving a set of well-characterized transcription factors. The synchronization of cells, controlled at the organismal level by a brain central clock, can be mimicked in vitro, pointing to the notion that all the cells are endowed with an autonomous time-keeping system. Metabolism undergoes circadian control, including the mitochondrial terminal catabolic pathways, culminating under aerobic conditions in the electron transfer to oxygen through the respiratory chain coupled to the ATP synthesis according to the oxidative phosphorylation chemiosmotic mechanism. In this study, we expanded upon previous isolated observations by utilizing multiple cell types, employing various synchronization protocols and different methodologies to measure mitochondrial oxygen consumption rates under conditions simulating various metabolic stressors. The results obtained clearly demonstrate that mitochondrial respiratory activity undergoes rhythmic oscillations in all tested cell types, regardless of their individual respiratory proficiency, indicating a phenomenon that can be generalized. However, notably, while primary cell types exhibited similar rhythmic respiratory profiles, cancer-derived cell lines displayed highly heterogeneous rhythmic changes. This observation confirms on the one hand the dysregulation of the circadian control of the oxidative metabolism observed in cancer, likely contributing to its development, and on the other hand underscores the necessity of personalized chronotherapy, which necessitates a detailed characterization of the cancer chronotype.

## 1. Introduction

Most of the organisms living on Earth are subjected to periodic and predictable environmental changes (light/darkness alternation, temperature fluctuations), which last approximately 24 h (circadian) according to the planet rotation period. During evolution, the organisms developed molecular time-keeping mechanisms, enabling them to anticipate the rhythmic geophysical changes, thereby acquiring competitive advantage [1,2].

In mammals, the synchronization of circadian rhythms in behavior and physiology is systemically controlled by the suprachiasmatic nucleus (SCN), located in the hypothalamus, and regulated by photic inputs conveyed by the retino-hypothalamic tract, which is one component of the optic nerve [3]. Circadian synchronization of cellular functions and biochemical processes accomplished by the SCN acts on peripheral tissues that utilize a common molecular clock machinery [4,5]. 

Mechanistically, rhythmicity occurs autonomously through a transcriptional–translational feedback loop (TTFL) involving a relatively small number of genes and related proteins constituting the “core” of the circadian clockwork [6]. A central role is played by the heterodimeric complex formed by CLOCK and BMAL1 transcription factors, which bind E-box regulatory sequences of genes including *Per* (period) and *Cry* (cryptochrome) genes, which encode proteins that heterodimerize, pass back in the nucleus and hinder CLOCK/BMAL1 transcriptional activity. In addition to this primary feedback loop, a secondary loop constituted by the orphan nuclear hormone receptors REV-ERBα and RORα operates, controlling negatively and positively the *BMAL1* transcription, respectively [6].

Most notably, CLOCK and BMAL1 prompt the expression of many clock-controlled genes (CCGs) encoding proteins and other transcription factors thereby driving primary and secondary transcriptomic waves involving thousands of downstream genes [7,8].

It has been estimated by transcriptomic analysis that in mammals 30–70% of the entire genome is under circadian clock control, depending on the considered tissue [7,8]. In this regard, mounting evidence indicates a tight time-dependent control of catabolic and anabolic cell metabolism [9]. Consistently, circadian disruption/dysregulation proved to have a negative impact on health, with increased metabolic risks and development of cardiovascular, cancer, immunological, neurological and psychiatric diseases as shown in animal models as well as in humans [10]. Intriguingly, factors affecting metabolism, such as physical exercise and diet, have been reported to influence in turn the circadian rhythms [11,12,13]. This suggests a complex reciprocal interplay between metabolism and biological rhythms that warrants deeper understanding in terms of molecular mechanisms [14]. 

Circadian timekeeping is an autonomous property of cells that even in the absence of external inputs is able to shape cyclically their metabolic profile, and several well-established procedures are available for in vitro synchronization of cultured cells [15,16,17]. Exploiting this feature, we showed in several studies that in vitro-synchronized cells exhibit a BMAL1-dependent oscillatory mitochondrial respiratory activity [18,19,20]. Interestingly, the period of the oscillating respiratory profile was significantly shorter than that expected for being truly circadian, resulting instead in ultradian rhythms (i.e., with a period of about 12–15 h). Ultradian rhythms have been, however, recently unraveled both in lower and higher organisms, suggesting the occurrence of different clockwork systems [21,22]. These can either be linked to the canonical circadian clock genes or can run autonomously in parallel to it. To better understand if such an ultradian activity was a specific property of the HepG2 cancer cell line that we routinely used in our previous investigations, we decided to extend our analysis to other cell types, either primary or cancer-derived lines, with the aim to obtain insights into the interplay between cell oxidative metabolism and biological clocks.

## 2. Results

### 2.1. Mitochondrial Respiration Undergoes Autonomous Oscillation in In Vitro Serum-Shocked Synchronized Cells

The assessment of time-resolved mitochondrial respiration in cultured cells following in vitro synchronization has been reported by several independent groups [18,19,23,24,25,26]. These studies unravel that mitochondria-related respiration undergoes a self-sustained autonomous oscillatory activity in the daytime scale. However, it must be pointed out that in most of these studies the respiratory activity is shown as the naive oxygen consumption rate (OCR), also referred to as the basal OCR. This is the result of a mixed pattern of substrates oxidation that depends on the metabolic profile of a given cell phenotype and on the composition of the buffered media in which they are kept growing or assayed. Basal OCR is coupled to the generation of a transmembrane electrochemical H^+^ gradient (∆*μ*H^+^, positive and acidic outside) provided by the redox-driven H^+^-translocation activity of the complexes I, III and IV of the respiratory chain [27,28,29]. This protonmotive force is utilized to drive exergonic reactions: firstly the synthesis of ATP by the H^+^ F_o_F_1_-ATP synthase but also the uptake of ions such as Ca^2+^ and charged respiratory metabolites such as pyruvate and glutamate [27,30,31]. However, the H^+^-gradient can also be unproductively dissipated because of a passive back-leak across the inner mitochondrial membrane or by uncoupling proteins-mediated back diffusion [32,33]. Importantly, the steady-state extent of the ∆*μ*H^+^ inhibits electrogenic electron transfer steps within the respiratory complexes and thus, in turn, controls the overall respiratory flux. Hence, the observed basal respiration is contributed, and independently modulated, by both an ATP-linked and a leak-linked OCR. A commonly utilized procedure to discriminate the two components of the basal OCR is to measure respiration in the presence of the ATP synthase-specific inhibitor oligomycin. Under this condition, the basal-OCR is significantly inhibited, the residual oligomycin-insensitive OCR is taken as a measure of the leak respiration and the difference between the basal OCR and the leak OCR is an indirect measure of the respiration productively leading to ATP synthesis. In the presence of a protonophore such as FCCP, which dissipates the protonmotive force and thereby unleashes its control on the electron transfer rate, the measured OCR is usually higher than that measured under basal conditions. This uncoupled OCR is referred to as the maximal attainable respiratory activity and the value exceeding the basal OCR is taken as a measure of the reserve capacity that can at the most be utilized under stressed energy-demanding metabolic conditions [34].

**Figure 1:**
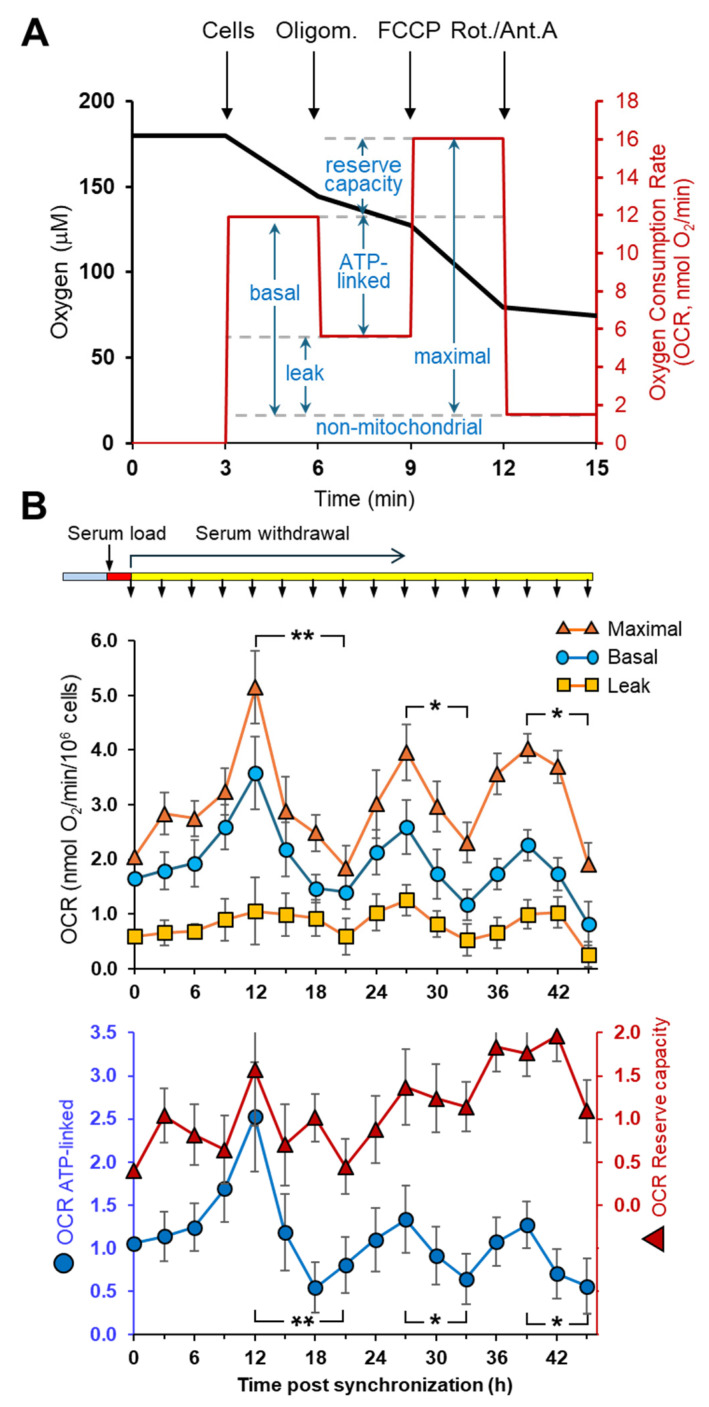
Analysis of mitochondrial respiratory activities in synchronized intact HepG2 cells. (**A**) Representative oximetric assay using the “mito stress” protocol as in [35]. Black trace refers to the changes in oxygen content; red trace refers to the rates of oxygen consumption. Where indicated, cells (5 × 10^6^ cells/mL), oligomycin (1 μg/mL), FCCP (0.4 μM) and rotenone (1 μM) plus antimycin A (1 μM) were added. The respirometric parameters discussed in the text are shown. (**B**) Effect of clock synchronization on the mitochondrial respiratory activities. HepG2 cells were synchronized by serum shock as described in Section 4 and are schematically shown on the top of the panel. The upper plot shows the basal, maximal and leak oxygen consumption rates (OCRs) of the cells collected at the indicated time points post-synchronization (modified from [18]). The lower plot shows the ATP-linked OCR and the respiratory reserve capacity estimated from the upper panel as shown in panel (**A**); for clarity, the right axis was scaled up. The averaged values (±SEM) for each time point, both in the upper and lower plot, of at least 10 biological replicates are shown. The statistical significance between the zenith and nadir of the three successive cycles for the basal, maximal and ATP-linked OCRs is shown: *, *p* < 0.05; **, *p* < 0.01.

Figure 1A shows a suite protocol often referred to as the sequential “mito stress test” [35,36,37], which can be used to extract all the respiration-linked parameters mentioned before and is executable with different instrumental devices such as the classical Clark-electrode or more recently developed electrodes containing [O_2_]-sensitive fluorescent probes [38]. In Figure 1A, addition of the complexes I and III inhibitors rotenone plus antimycin A, respectively, is also shown to assess the non-mitochondrial linked respiration. Subtracting this last OCR from those previously recorded would return the bona fide activities of the mitochondrial respiratory chain.

Figure 1B shows the results attained when the described “mito stress test” was applied to measure the respirometric parameters in the hepatocellular cancer cell line HepG2 subjected to in vitro synchronization of the circadian clockwork [18]. The synchronization protocol shown for this set of measurements relied on the serum-shock procedure, consisting in exposing the cell culture to a high concentration of serum (typically 50%) for 2 h, whose removal was shown to synchronize the expression of the core clock genes in a major part of the cell population [15]. At regular intervals of time post-synchronization (i.e., every three hours up to 45 h post the synchronization start), cells were collected and analyzed in suspension by electrodic oxymetry following the above-described mito stress test protocol. The values reported in the upper panel are the raw values of the OCRs corrected for the Rotenone/Antimycin A-insensitive OCR. It can be appreciated that the basal OCR displayed a clear spontaneous oscillatory activity, with three peaks in the chosen time window of observation. A similar oscillatory profile was also observed for the OCR leak (i.e., in the presence of oligomycin) and for the maximal OCR (i.e., in the presence of FCCP) although, as expected, at every time point the activity was significantly lower and larger, respectively. The lower panel shows the ATP-linked OCRs and the reserve respiratory capacities as computed from the raw data set. It can be noted that the ATP-linked OCR maintains an oscillatory profile, as does the basal OCR (see ahead), though the relative height of the peaks slowly declines at later time points, likely because of a progressive loss of synchronization. Conversely, the spare capacity apparently does not exhibit significantly oscillatory changes throughout the experimental time window. Changing the respiratory substrate (i.e., substituting glucose with galactose in the culturing and assay medium) or utilizing different synchronization protocols such as a short incubation with dexamethasone, followed by wash-out, similarly resulted in rhythmic variations in the mitochondrial respiration [18]. 

### 2.2. Rhythmic Mitochondrial Respiration Depends on Bmal1 Expression

**Figure 2:**
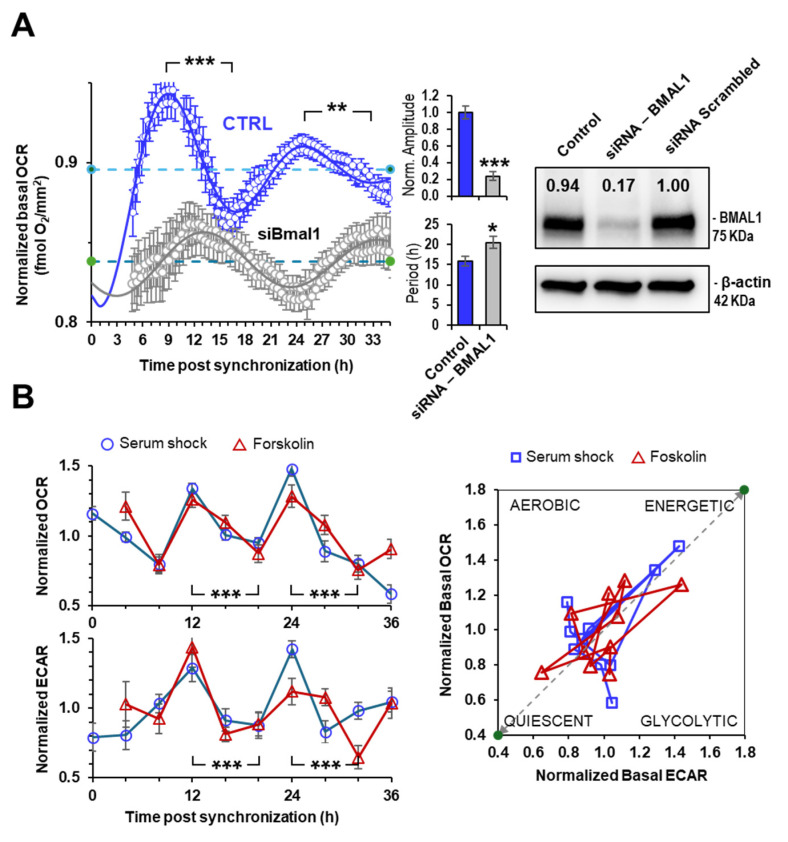
Measurement of respiratory activities of synchronized cells by non-polarimetric methodologies. (**A**) Representative real-time monitoring of the basal OCR on adherent HepG2 cells following serum-shock synchronization by Resipher technology. The blue points (averages of six technical replicates ± SEM) refer to control cells, and the grey points to Bmal1-silenced cells, shown in the western blot on the right side. The bar graphs show the normalized amplitude and the period of the oscillatory OCR profiles in control and siRNA-BMAL1 cells as computed by the Cosinor best-fitting procedure (see text) and refer to three biological replicates: *, *p* < 0.05; **, *p* < 0.01; ***, *p* < 0.001. (**B**) Representative OCR (**upper panel**) and ECAR (**lower panel**) time-resolved profiles in synchronized NHDF assayed by Seahorse technology (data for serum-shock synchronized cells are modified from [23]). Synchronization was performed by either serum shock (blue profiles) or forskolin treatment (red profiles) as detailed in Materials and Methods. The points are averages (±SEM) of two biological replicates (four technical replicates/biological replicates) under each condition and were normalized to the mean value of all the time points recorded during the experiment; the statistical significance between the zenith and nadir of two consecutive cycles is shown: ***, *p* < 0.001. The panel on the right shows the bioenergetic plot correlating the normalized OCRs and ECARs from the data indicated in the left panels.

When the above-reported analysis was carried out in non-synchronized HepG2 cells, the oscillatory respiratory profile was remarkably dampened [18] and, most notably, the oscillatory OCR observed in synchronized cells correlated with the expression of the core clock master gene *Bmal1* [18]. Consistently, the silencing of Bmal1 markedly inhibited the robustness of the oscillating respiration in synchronized cells [18,19]. These results were fully confirmed by following in real-time (i.e., every 15 min) the cellular respiratory activity directly in the microplate, utilizing a recently established new solid-phase optical oxygen-sensor detection system (i.e., Resipher) (Figure 2A). Eighty percent silencing of the Bmal1 expression attained by the siRNA-based approach resulted in a substantial depression/deregulation of the rhythmic activity of the mitochondrial respiration, resulting in a reduction in the oscillatory amplitude and elongation of the oscillatory profile (Figure 2A).

Interestingly, when the mitochondrial respiration was followed by the Seahorse platform alongside glycolysis in synchronized cells both the metabolic fluxes displayed an in-phase similar oscillatory profile. This is shown in Figure 2B when, to note, primary human dermal fibroblasts (NHDF) were utilized and synchronization was achieved, in addition to serum shock, by a short-time exposure of the cells to forskolin (an additional established protocol of synchronization [16]). Consequently, the “bioenergetic” plot correlating the basal OCR and basal glycolysis at each time point resulted in an oscillatory pattern alternating between an energetic and a quiescent state (Figure 2B).

Overall, the so-far reported observations clearly indicated that both a primary and a cancer-derived cell type are endowed with an autonomously controlled time-keeping mechanism regulating their basal metabolism. To further characterize this process, we decided to analyze comparatively different cell samples that have been used over time in our lab. The comparison was performed on the following cell types: primary cells (NHDF), primary immortalized cells (HAEC), tumor cells (HepG2, HeLa, U2OS, Cal27, SCC9) and tumor and metastatic cells from the same tissue (SW480 and SW620). For Hela and U2OS, the time-resolved respirometric analysis was performed by the mito stress test, as for HepG2 and NHDF; for all the other cell types, only the basal OCRs corrected for the rotenone-insensitive activity were available. This is because the latter were analyzed in research projects where a rapid screening focusing solely on the basal OCR was needed.

### 2.3. The Autonomous Oscillatory Profile of Mitochondrial Respiration Differs among Different Cell Types

**Figure 3:**
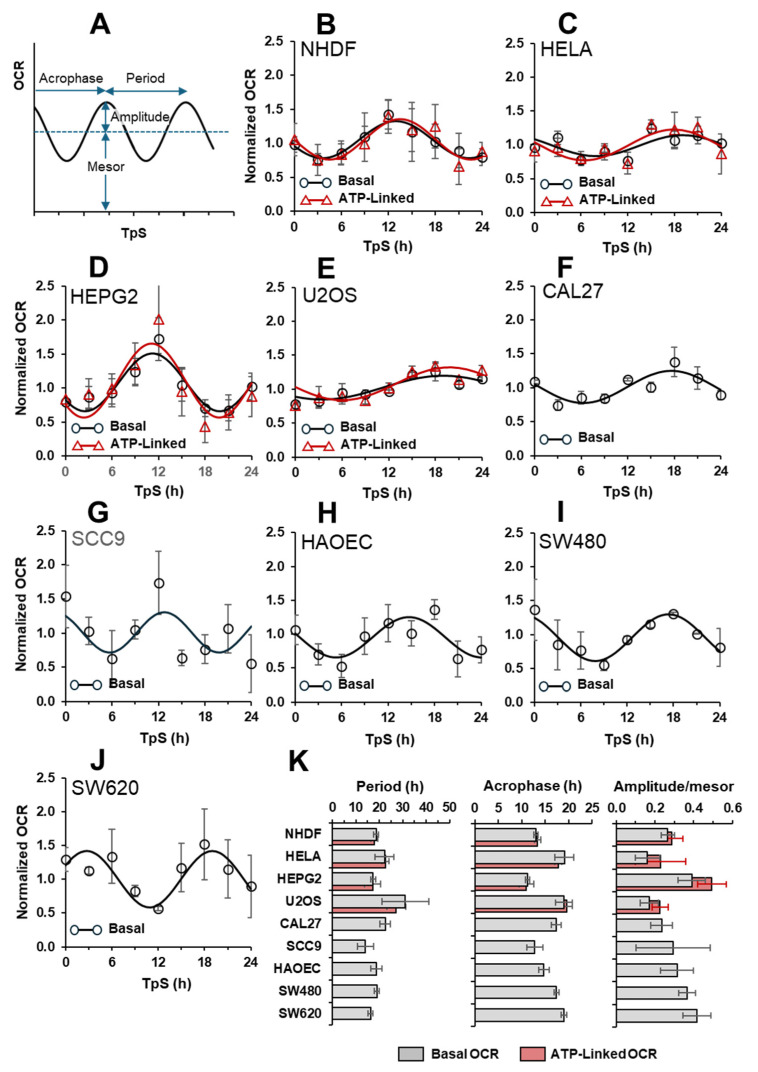
Cosinor-mediated best-fitting of the OCR profiles in a variety of clock genes synchronized primary cells and cancer-derived cell lines. (**A**) Scheme showing the parameters of the Cosinor equation (see Section 4) in the context of a generic oscillatory rhythm. (**B**–**J**) Best fit of the experimental basal OCRs, measured polarographically in the indicated serum-shock synchronized cells. (**B**–**E**) The ATP-linked OCRs. The points are normalized to the mean value of all the time points recorded during the time course and are the average (±SEM) of at least three independent biological replicates. The continuous lines are the best fit of the experimental points using the Cosinor model with an χ^2^ < 0.05. (**K**) Bar graphs showing the triads of Cosinor parameters (i.e., period, acrophase and amplitude/mesor) of the best-fitting curves are shown in panels (**B**–**J**). (The order in which the cells are shown reflects a progressively decreased value of mitochondrial resting respiration as reported in the next figure, Figure 4).

Figure 3 shows the respiratory activities of all the above-mentioned cell types recorded in the first 24 h every 3 h post serum-shock synchronization. As the resting/basal OCR was significantly different among the cell samples (see ahead), the values reported at each time point were normalized to the average value of the data set over the 24 h of the experiment. The average value was not significantly different from that measured in non-synchronized cells. Hence, the raw data were best-fitted by non-linear regression analysis using the Cosinor equation [39], which provides quantitative estimation of the parameters shaping a regular oscillatory profile. These parameters are as follows: mesor, the midline statistic of rhythm average value; amplitude, a measure of half the extent of variation within a cycle; period, duration of one cycle; and acrophase, a measure of the time of overall high values recurring in each cycle. In our analysis we used the amplitude/mesor ratio as a normalizing feature irrespective of the respiratory phenotype among the different cell samples. 

As shown in the multi-panel of Figure 3, all the cell samples tested exhibited a clear oscillating OCR pattern following in vitro synchronization. To note, in the four cell types where the best fit of both the normalized basal OCRs and normalized ATP-linked OCRs was performed, no significant difference was observed for the given cell type either for the overall oscillatory profile or the fitting Cosinor parameters. This is of bioenergetics relevance since we can utilize, with acceptable confidence, the rhythmic parameters attained by fitting the basal OCR as a bona fide quantification of the oscillatory activity of the cell respiration linked to ATP synthesis. 

**Figure 4:**
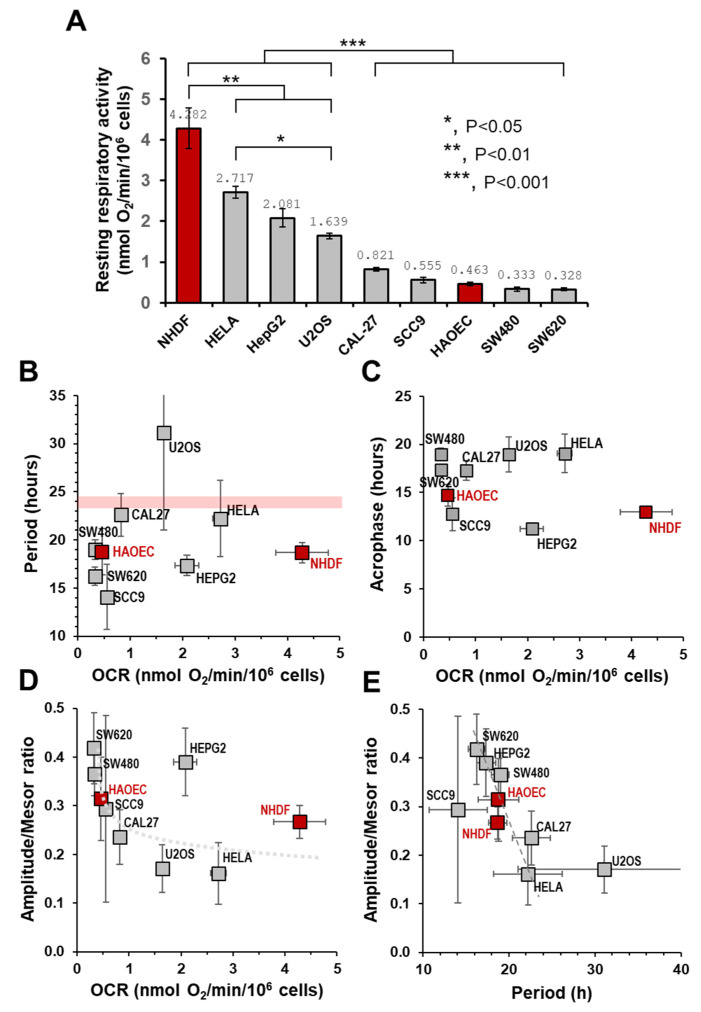
Correlations between cellular mitochondrial respiratory proficiency and Cosinor-related parameters. (**A**) The resting/basal OCR is shown for all the indicated cell types used in this study. The values shown refer to the respiratory activity under non-synchronized conditions (not statistically different from the averaged values of the basal OCRs measured during a time course experiment post-synchronization) and are the average (±SEM) of at least five independent biological replicates for each cell type. The statistical analysis of the differences is also shown; for clarity, the statistically significant differences are also indicated between groups of cells. (**B**–**D**) Plots correlating to the respiratory activities of the cell types are shown in panel (**A**), with the Cosinor parameters (i.e., period, acrophase and amplitude/mesor) best fitting the temporal profiles post-synchronization of the same cell types shown in Figure 3K. The light red line in (**B**) indicates the position in the plot of a canonical circadian oscillation. (**E**) Correlation plot between the Cosinor parameters period and amplitude/mesor best fitting the temporal profiles of the normalized OCRs in the indicated synchronized cells. The red bars in (**A**) and red squares in (**B**–**E**) indicate non-tumor-derived primary cells; the grey bars and squares indicate tumor-derived cell lines.

All the cell types tested displayed an autonomous rhythmic mitochondrial normalized OCR, though a heterogeneous combination of the best-fitting Cosinor parameters was clearly observed. As the cohort of cultured cells displayed large differences in the basal/resting OCR (Figure 4A)—likely reflecting specific respiratory phenotypes—we sought to correlate it with one or the other of the Cosinor parameters. As shown in Figure 4B, no correlation was found between the extent of the period (ranging from 14 to 31 h) and the basal OCR. However, it can be noted that, except for HeLa and CAL27 cell lines, whose period approached the circadian period, all the other cell types, irrespective of them being primary or tumor-derived, displayed a significantly lower period (i.e., an ultradian rhythm: see [40,41]). An exception to this was the case of U2OS, which exhibited a period longer than 24 h. No correlation was found between the acrophase and the basal respiration (Figure 4C). Also, in this case the values where the positive peak appeared were spread among the different cell samples from 10 to 20 h post-synchronization. When plotting the normalized amplitudes as a function of respiration, apart from the HepG2 cell line, an apparent inverse correlation was observed with an exponential-like profile (Figure 4D). Consequently, at the lower OCR the relative amplitude of the oscillation was increased from about 15% to 43%, as referred to as the mesor of the cell type. Irrespective of the basal respiratory phenotype, a clearer inverse correlation was observed when the relative amplitudes were plotted as a function of the period for each cell type tested (Figure 4E). 

**Figure 5:**
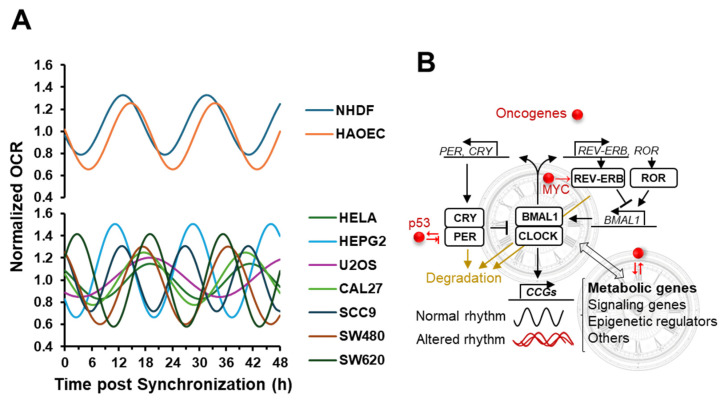
Comparative analysis of the temporal profiles of the mitochondrial respiration in different synchronized cell types. (**A**) The synchronization-mediated oscillatory OCR profiles of primary cells (upper plot) were compared with those of cancer-derived cell lines. For simplicity, only the Cosinor curves best fitting the experimental data points for the different cell types, shown in Figure 3B–J, are displayed, and extended to 48 h. (**B**) Schematic drawing of the circadian core clock transcriptional and translational feedback loop and its intertwining with metabolism as well as oncogenes. The red dot indicates oncogenes, with specific mention of those documented to interact with components of the circadian clockwork (i.e., MYC, p53). Oncogenes that regulate various cellular processes, including the expression of genes encoding metabolism-related proteins, are not explicitly marked. The double-headed arrow illustrates a reciprocal interplay between the circadian clockwork and cellular metabolism (see text for explanation).

A comparison of the Cosinor-derived curves fitting the respiratory profiles of the two primary cell types (NHDF and HAEC) with those of the cancer cell lines resulted in a notable distinction. While the former exhibited almost superimposable patterns, the latter displayed a heterogeneous assembly characterized by phase/period misalignment and changes in relative amplitude (Figure 5A).

## 3. Discussion

Oscillatory rhythms during the daytime modulate various physiological processes at both organismal and cellular levels. The circadian clock genes, operating through a transcription–translation feedback loop, constitute the most well-characterized time-keeping mechanism, orchestrating autonomous rhythmic outputs. According to time-resolved transcriptomic, proteomic and metabolomic analyses, 50% to 80% of the mammalian genome is subject to circadian control, varying depending on organ and cell phenotype [7,8]. Consequently, numerous metabolic pathways exhibit functional oscillations throughout the day [9,11,12,13,14]. Notably, mitochondrial terminal metabolism, crucial for bioenergetics and the ATP provision necessary for cellular homeostasis, is no exception [26,42,43,44].

Assessing the efficiency of electron transfer in the mitochondrial respiratory chain, needed for ATP synthesis, can be achieved by measuring the oxygen consumption rate in isolated mitochondria or intact cultured cells [34]. The traditional polarographic method, while valuable, has been complemented by recent technologies, making cellular respiration measurement increasingly accessible [36,38]. Coupled with the ability to replicate circadian clockwork in vitro through established synchronization protocols [45], this represents a relatively straightforward experimental procedure for evaluating a cell’s energetic chronotype.

In previous studies from our laboratory, it has been reported that the cell respiration largely attributable to the mitochondrial electron transfer chain undergoes an autonomous robust oscillatory activity following in vitro synchronization. This is not dependent on the respiratory carbon source, the synchronization protocol or the method of measurement. Notably, this process was observed both in human primary cells (i.e., NHDF—fibroblasts) and cancer-derived cell lines (i.e., HepG2—hepatocellular carcinoma) and is linked to the expression of *Bmal1*, the master gene of the circadian clockwork [18,19,23]. Mechanistically, we demonstrated that the alternating activity of the respiratory chain was dependent on the acetylation state of a subunit of the NADH dehydrogenase (complex I), which was related to the NAMPT-mediated synthesis of NAD^+^ and the activity of sirtuins, both, in turn, rhythmically changing under the control of the circadian clockwork [18] (see also [46,47]). 

Moreover, we found that an oscillatory mitochondrial Ca^2+^-mediated reversible phosphorylation of the pyruvate dehydrogenase complex was responsible for a rhythmic pyruvate oxidation [20]. Therefore, the rhythmic oxidative phosphorylation was on the one hand dependent on the upstream availability of oxidizable substrates and on the other hand on the contemporary downstream functional activation of the respiratory chain complex I. Several studies have shown the circadian expression of nuclear genes coding for the mitochondrial OxPhos system and dynamics [24,48,49,50], whereas a few others have occasionally reported rhythmic changes in the expression of mitochondrial DNA-encoded genes [7,21,48]. 

In Figure 1A,B, we detailed how to extract the largest amount of information from an oxygraphic assay mimicking different physiological conditions [34,35,36]. Importantly, the observed oscillatory pattern obtained for the basal respiration was maintained when correction was made for the oligomycin-insensitive OCR, which is a parameter better reflecting the oxygen consumed as functionally linked to the synthesis of ATP. The reserve capacity of the OCRs, on the other hand, did not unveiled an overt oscillatory profile. This was since the maximal activity paralleled the basal activity so that their difference was roughly constant. This is consistent with a modulation in the catalytic activity of complex I exerted reversibly at the post-translation level [18]. 

In Figure 2A, we applied a recently developed methodology using a fluorescent O_2_-sensitive probe to measure in real-time the OCR directly in the microplate following in vitro synchronization (i.e., without the need of detaching and suspending the cells from the culturing plate). The result attained clearly confirmed what was observed by traditional oxygraphy on suspended cells. Importantly, the observed respiratory profile was largely dampened in BMAL1-silenced cells. When the respiratory flux was followed along with the glycolytic flux, using the Seahorse technology, the time-resolved profiles of the two fluxes displayed in-phase oscillatory activities, thereby unveiling no compensatory up-regulation of one flux when the other was down-regulated (Figure 2B). This is consistent with a robust rhythmic variation in the cellular ATP content that we reported in [20] and with the consequent alternation of the synchronized cells between an energized and an almost-quiescent metabolic state (Figure 2B). 

This study aimed to reinforce the notion that the self-sustaining rhythmic respiration was a general phenomenon. To this purpose, we selected as compared with previous works from our group an additional primary cell type and more tumor-derived cell lines and analyzed their time-resolved respiratory activity under similar conditions. The basal cell respiration was variable among the different cell type regardless of whether they were primary or cancer-derived lines; moreover, for some of the cell types the mito stress test was applied, whereas for others only the basal OCRs corrected for the rotenone-insensitive activity were available. Thus, to make the data sets homogeneous the time-resolved basal OCRs were plotted as normalized to the average value of all the OCRs measured during the experimental time course; the same was made for the ATP-linked OCRs (computed as in Figure 1) when available. Hence, the experimental data sets were fitted with the Cosinor equation, enabling to quantify the basic parameters shaping the oscillatory profiles [39]. Notably, no statistically significant differences were attained with the Cosinor parameters (i.e., period, acrophase and amplitude/mesor) describing the basal and ATP-linked OCR profiles related to a given cell type. This is important because it enabled utilizing the simple basal OCRs (normalized to the averages value) as a *bona fide* indication of the bioenergetically functional respiratory activity. 

The analysis of the data shown in Figure 3 confirmed the occurrence of an autonomous rhythmic mitochondrial respiratory activity in practically all the synchronized cellular samples tested, thereby reinforcing the notion that this is a general phenomenon. On the other hand, comparison of the Cosinor parameters yields a highly heterogeneous picture. The attempt to find a correlation between one or the other of the Cosinor parameters with the different respiratory phenotypes failed to provide significant results. The sole relevant inverse correlation was between the relative amplitude of the oscillation and its period for a given cell type, but it was independent of the respiratory rate exhibited by the cell type. 

However, and intriguingly, when comparing the Cosinor-derived curves fitting the respiratory profiles of the two primary cell types (NHDF and HAEC) with those of the cancer cell lines, a notable distinction emerged. While the former exhibited almost superimposable patterns, the latter displayed a heterogeneous assembly characterized by phase/period misalignment and changes in relative amplitude (Figure 5A).

The heterogeneity of circadian rhythms in cancer cells is a fascinating and clinically significant aspect of tumor biology [51,52,53]. While the circadian clock regulates various physiological processes in healthy cells, including metabolism, proliferation and DNA repair, cancer cells often exhibit disrupted or altered circadian rhythms [54]. This heterogeneity in circadian rhythms among cancer cells can have profound implications for tumor growth, treatment response and patient outcomes. 

A complex interdependent relationship of oncogenesis, metabolism and the circadian clock is emerging [55]. Alterations in the physiological circadian rhythms such as those occurring in chronic sleep–wake disorders have been linked to a higher risk of developing cancers of the breast, colon, ovaries and prostate [56]. Accordingly, mutations in molecular clock genes have been documented across many different types of cancer (though with less than 20% incidence per tumor type) [57,58,59]. Therefore, it has been suggested that the clock may function as a tumor suppressor. Many proto-oncogenes as well as tumor suppressors are normally under circadian control [60] and so deregulation of the clock-mediated oscillation can lead to constitutive alterations in their expression.

The consequences on the metabolic phenotype resulting from the interaction between oncogenes and the circadian clock in the carcinogenesis context become even more intricate considering that both catabolic and anabolic pathways are largely controlled by both oncogenes and circadian clock genes. Moreover, the latter are reciprocally modulated by metabolic products. Indeed, the clock-controlled NAMPT causes oscillating availability of NAD^+^, the substrate of sirtuins that opposes the acetyltransferase activity of CLOCK deacetylating BMAL1, PER and histones, thus resulting in modulation of both the phase and amplitude of the circadian gene oscillation [46,61]. It cannot be overlooked that NAD^+^ is also an essential metabolic redox cofactor and that the NAD^+^/NADH ratio is shaped by the metabolic profile of a cell. AMPK is another linker between metabolism and the circadian clock. Indeed, activated by the oncogene kinase LKB1, AMPK is a sensor of the metabolic state of the cell in terms of the AMP/ATP ratio, and following further activation by Sirt1 it negatively modulates the proteolytic degradation of PER and CRY, leading to up-regulation of clock-controlled genes [62]. 

Consistent with the above-mentioned notions but more focalized on the mitochondrial respiratory metabolism, our group demonstrated in the past that either pharmacological and genetic inhibition of the respiratory chain, as well as inhibition of the oscillatory entry of Ca^2+^ into mitochondria, led to a marked specific deregulation in the rhythmic expression of the core clock genes [19,20]. 

Overall, the heterogeneity of the respiratory profile in the synchronized cancer-derived cell lines, shown in Figure 5A, can therefore be rationalized because of the intertwined interactions between oncogenes and the circadian clockwork, whose metabolic outcome would specifically depend on the genetic background of a given cancer cell (Figure 5B). 

A key point that emerged from the comparative analysis presented here is the variability in the period length of the oscillatory profiles of mitochondrial respiration. These periods range from values close to the canonical 24 h circadian rhythms to both shorter and, in one case, longer durations (see Figure 4B). Although the observational time window reported in this study for the serial measurements in cells post-synchronization is 24 h, we are confident that they are representative of the major features of the autonomous time-keeping machinery. This confidence is based on longer time series analyses in cells such as HepG2 and NHDF, which resulted in rhythmic parameters similar to those reported here, particularly concerning period length. This observation applies to measurements of OCRs and other mitochondria-related functions such as ATP levels, NAD^+^ production, ROS generation [18,19,20] and the expression of factors controlling mitochondrial biogenesis and dynamics (unpublished results). Additionally, the Cosinor-based non-linear regression analysis yielded statistically robust fittings of the experimental time-resolved data.

Several mechanisms have been proposed to account for ultradian rhythms (i.e., periods in the 12–15 h range) driven by circadian clock genes [40] or alternative time-keeping systems integrated with them [21,22,63,64]. Although we cannot provide mechanistic insights into these non-circadian rhythms, the results presented here suggest a dysregulation or a different regulation of rhythmic mitochondrial respiration depending on the cellular phenotype or genotype.

The heterogeneity of circadian rhythms in cancer cells has implications for chronotherapy, which involves administering drugs at specific times in the day to maximize efficacy and minimize toxicity [65,66]. The effectiveness of chronotherapy depends on the synchronization of drug administration with the circadian rhythms of the tumor cells. However, the heterogeneity of circadian rhythms among different tumors and even within the same tumor can complicate the timing of drug delivery and may require personalized approaches based on individual cellular circadian profiles.

Despite the widely overemphasized “Warburg effect”, mitochondrial oxidative phosphorylation is emerging as a crucial aspect of the cancer bioenergetic phenotype, with varying impacts depending on the specific cancer type and its developmental stage [67,68]. Consequently, targeting mitochondrial oxidative metabolism in cancer is being explored as an alternative or adjunctive therapeutic approach [69,70]. However, the findings of this study, which highlight the rhythmic nature of the cellular bioenergetics, suggest that for an antimetabolite drug targeting mitochondrial respiration to be effective it should be administered before the onset of respiratory peaks to attenuate them and induce a bioenergetic crisis. In addition to pharmacological treatments, a time-controlled dietary regimen might also be exploited [53,71,72]. Indeed, it has been shown that fasting as well as time-restricted feeding proved to impact on the circadian regulation of cancer metabolism, reducing the risk of its development as well as slowing its growth [73,74]. Understanding the period of oscillating respiration for a given cancer chronotype could certainly aid in determining the optimal timing of treatment.

## 4. Materials and Methods

### 4.1. Cells and Culture Conditions 

All the cell types used in the present study (see Table 1) were purchased from ATCC. Cell cultures were maintained in the DMEM-based specific medium, as reported by the manufacturer, at 37 °C in the presence of 5% CO_2_ supplemented with 10 mM Hepes, 10% inactivated fetal bovine serum (FBS), 2 mM glutamine, 100 U/mL of penicillin and 100 μg/mL of streptomycin. All the cells were assayed at passage < 15.

### 4.2. In Vitro Circadian Rhythms Synchronization

Clock genes synchronization in cell cultures was achieved by different protocols as described in [15,16]. Briefly, for serum shock, 3 × 10^6^ cells/dish were treated with a high concentration of serum (i.e., 50%) for 2 h followed by a change with a serum-free medium. Alternatively, the cells were synchronized by incubation either with 0.1 μM dexamethasone (directly added to the culturing medium) for 30 min or with 10 μM forskolin (directly added to the culturing medium) for 15 min; thereafter, the synchronizers were washed out by replenishing cells with DMEM (+10% FBS). Cells were harvested and assayed at the different time points post-synchronization as indicated in the text/figures. Cell cultures were typically utilized at a passage number below 15 and at a confluence of 80–85%.

### 4.3. Respirometric Measurements

The oxygen consumption rates (OCRs) were measured in intact cells either by Clark electrode-based polarography or by an O_2_-sensitive fluorescent probe. For polarographic measurements, cultured cells were gently detached from the dish by trypsinization, washed in PBS, harvested by centrifugation at 500× *g* for 5 min and immediately assessed for O_2_ consumption with either an Oxygraph-2 k (Oroboros Instruments, Innsbruck, Austria) or Hansatech oximeter (Hansatech Instruments, Narborough Rd, Pentney, King’s Lynn PE32 1JL, Regno Unito, UK). About 8–10 × 10^6^ viable cells/mL were assayed in their specific culture medium at 37 °C; after attainment of a stationary endogenous substrate-sustained basal oxygen consumption rate (basal OCR), 2 μg/mL of the ATP-synthase inhibitor oligomycin was added (leak OCR) followed by addition of 0.3 μM of the uncoupler carbonilcyanide p-triflouromethoxyphenylhydrazone (FCCP) (maximal OCR). The rates of oxygen consumption were corrected for 2 μM antimycin A + 2 μM rotenone-insensitive respiration and normalized to the initial cell number; the ATP-linked OCR was estimated from the difference between the basal OCR and leak OCR and the OCR reserve capacity from the difference between the maximal OCR and basal OCR. Alternatively, the OCR was measured in real-time in adherent cells by Resipher system technology (Lucid Scientific Inc. Atlanta, GA, USA) following the manufacturer’s protocol; 20,000 cells/100 μL/well were plated in the 96-well cartridge (in technical triplicates) and placed in the CO_2_ incubator at 37 °C with measurements of the basal OCR taken every 15 min for at least 36 consecutive hours; the first 3–4 h were not taken into account because of temperature equilibration. For measurement of the metabolic fluxes, the Seahorse technology was used as in [23]. Briefly, distinct groups of cell samples, plated in the multi-well cartridge, were subjected to the synchronization protocols following a 4 h out-of-phase time schedule enabling assessment of all the samples in the same experimental session. The OCR and extra-cellular acidification rate (ECAR) were measured simultaneously in adherent cells with a XF96 Extracellular Flux Analyzer (Seahorse Bioscience, Billerica, MA, USA). After replacing the growth medium with 180 μL of bicarbonate-free DMEM supplemented with 10 mM glucose 2 mM L-glutamine and 1 mM sodium pyruvate pre-warmed at 37 °C, cells were preincubated for 45 min before starting the assay procedure. After measuring the basal OCR and ECAR, oligomycin (1 μM), carbonyl cyanide m-chlorophenylhydrazone (0.5 μM), rotenone + antimycin A (1 μM + 1 μM) and 2-deoxy-glucose were injected into each well sequentially to assess the leak, maximal and non-mitochondrial OCR as described above. For the ECAR (an indirect estimate of glycolysis), addition of oligomycin caused an increase in the basal value and it is taken as a measure of the maximal glycolytic capacity (no significant changes were observed upon the successive addition of carbonyl cyanide m-chlorophenylhydrazone and rotenone plus antimycin A); 2-deoxy-glucose caused inhibition of the glycolytic flux and the residual ECAR was subtracted from the basal ECAR. The values were normalized to protein content in each well, determined with a BCA assay.

### 4.4. BMAL1 Silencing in HepG2 Cells

BMAL1-specific siRNA was purchased from Thermo Fisher (Mission Pre-designed siRNA). HepG2 cells were seeded on 60 mm dishes and at 30–50% confluence were transiently transfected with the BMAL1-specific siRNA diluted in Opti-MEM using Lipofectamine^®^ 3000 Transfection Reagent (Invitrogen, Waltham, MA, USA) according to the manufacturer’s protocol. After 18–20 h of incubation at 37 °C, the transfection medium was replaced with complete medium containing 10% FBS and after 72 h (where the maximal efficiency of transfection was reached) cells were collected and assayed.

### 4.5. Western Blotting Analysis

Aliquots, containing 40 μg of protein extract from cell lysate (from 5 × 10^6^ cells), were subjected to SDS-PAGE (12% acrylamide) and transferred to a PVDF membrane (Bio-Rad Laboratories, Hercules, CA, USA) using a Trans Blot Turbo System. Then, membranes were probed with the following antibodies: anti-*BMAL1*, a primary rabbit polyclonal Ab (14020 Cell Signaling, dil. 1:1000, Danvers, MA, USA); a HRP-conjugated anti-rabbit IgG as a secondary Ab (31460 Thermo Scientific, dil. 1:10,000, Waltham, MA, USA); anti-β-actin (1:10,000 mouse Ab from Sigma, St. Louis, MO, USA); and a peroxidase-conjugated anti-mouse secondary antibody (Bio-Rad, 1:10,000). The signals were developed using the enhanced chemiluminescence kit (Clarity Western ECL Substrate, Bio-Rad) acquired with the ChemiDoc imaging system XRS + (Bio-Rad), and then analyzed using ImageJ software (Version 1.54j 2024) https://imagej.net/ij/ accessed on 17 June 2024.

### 4.6. Cosinor Analysis

The temporally resolved data of cellular respiration (OCRs) after in vitro synchronization were submitted to non-linear regression analysis by the Cosinor equation [39]: f(x) = M + Acos(2πx/P + *ϕ*), where f(t) = OCR; x = time post-synchronization; M = mesor (Midline Statistic Of Rhythm, a rhythm-adjusted mean); A = amplitude (a measure of half the extent of predictable variation within a cycle); P = period (duration of one cycle); and *ϕ* = acrophase (a measure of the time of overall high values recurring in each cycle, corresponding to the time post-synchronization when the first peak appears). The above-described simple equation was utilized to fit OCRs measured within 24 h post-synchronization; for longer times post-synchronization, the equation was modified as follows: f(x) = M + Ae^vt^cos(2πx/P + *ϕ*) + st, where v = variation in the amplitude (A) in subsequent cycles and s = slope (gradual slant of the overall data set profile). The analysis was carried out with GraFit version 7 (Erithacus Software) by initial approximation of the parameters; several fitting routines/cycles were allowed till reaching a χ^2^ < 0.05. 

## Figures and Tables

**Table 1:** Primary cells and seven cancer-derived cell lines that were assayed in this study; all the cell lines were from primary tumors, except SW620, which was from a metastatic source.

Name	Organ	Type	Mutated Genes
HAEC	Aorta	Endothelial	N.A.
NHDF	Skin	Fibroblast	N.A.
Cal27	Tongue	Squamous cell carcinoma	TP53, TERT, CDKN2A, KMT2A, LRBP1
Hela	Cervix	Adenocarcinoma	HPV E6 E7, MED1, ERBB3 CASP8, HLA-A, TGFBR2
HepG2	Liver	Epithelial carcinoma	TP53, TERT promoter, CTNNB1
SCC9	Tongue	Squamous cell carcinoma	TP53, TERT, CDKN2A, KMT2A, LRBP1, HIF1A
SW480	Colon	Adenocarcinoma	APC, TP53, ERBB2, KRAS
SW620	Colon	Adenocarcinoma (met)	APC, TP53, KRAS
U2OS	Bone	Epithelial osteosarcoma	RB, TP53, RECQLC2, RECQLC3, RECQLC4

## Data Availability

The raw data supporting the conclusions of this article will be made available by the authors on request.
